# Current update on theranostic roles of cyclophilin A in kidney diseases

**DOI:** 10.7150/thno.72948

**Published:** 2022-05-13

**Authors:** Sudarat Hadpech, Visith Thongboonkerd

**Affiliations:** Medical Proteomics Unit, Office for Research and Development, Faculty of Medicine Siriraj Hospital, Mahidol University, Bangkok, Thailand

**Keywords:** AKI, Biomarker, CKD, Diabetic nephropathy, Nephrotoxicity, PPIA, Renal fibrosis, Therapeutics

## Abstract

Cyclophilin A (CyPA) or peptidylprolyl isomerase A (PPIA), an immunophilin with peptidyl-prolyl *cis/trans* isomerase (PPIase) activity, is an abundant cellular protein widely expressed across various cell types and tissues, including the kidney. Expression of CyPA in the kidney is relatively higher in proximal tubular epithelial cells than others along the nephron. Alterations in expression and secretion of CyPA play important roles in physiological processes and pathophysiology of several diseases affecting the kidney. Herein, we provide a brief overview of CyPA structural biology and present the current update on its theranostic roles in various kidney diseases, including diabetic nephropathy, acute kidney injury, chronic kidney disease, renal fibrosis, and nephrotoxicity associated with organ transplantation. Notably, the diagnostic/prognostic role for urinary CyPA in several of these kidney diseases is promising. Finally, future perspectives on the CyPA research, especially targeting CyPA for therapeutics, are discussed. A comprehensive understanding of the theranostic roles of CyPA in kidney diseases is expected to provide novel insights into the design of new therapeutic interventions and prevention strategies.

## 1. Introduction

Cyclophilin A (CyPA), also known as peptidylprolyl isomerase A (PPIA), is one among several proteins in a family with peptidyl-prolyl *cis/trans* isomerase (PPIase) activity. The PPIase activity was first identified by Fischer et al. 1 in 1984 as a catalytic enzyme for conversion of *cis* and *trans* isomers of proline imidic peptide bonds**.** In the same year, Handschumacher et al. 2 firstly reported that an 18-kDa protein from bovine thymocytes serves as an intracellular receptor for an immunosuppressive agent, cyclosporin A (CsA). Therefore, this CsA-binding protein has been named as CyPA 2. By the end of the 1980s, PPIase and cyclophilin were found to be the same molecule 3, 4. However, it has been documented that the immunosuppressive effect of CsA is unrelated to the isomerase activity of PPIase but rather occurs via formation of the CsA**-**CyPA complex, which inhibits calcineurin activity, leading to suppression of T-cell activation through nuclear factor of the activated T-cells (NFAT) pathway 5-7. CyPA can be intracellularly and extracellularly expressed in many cell types, e.g., vascular smooth muscle cells (VSMCs), endothelial cells, macrophages, and kidney cells 8, 9. As a result, CyPA serves as a multifunctional protein 8, 9. Although the roles for CyPA have been well documented in several diseases and conditions, its roles in kidney diseases remained not well understood and under-investigated**.** In this article, we therefore provide a brief overview of the CyPA structural biology and update the current knowledge on its theranostic roles in various kidney diseases.

## 2. A brief overview of CyPA structural biology

**Figure 1:**
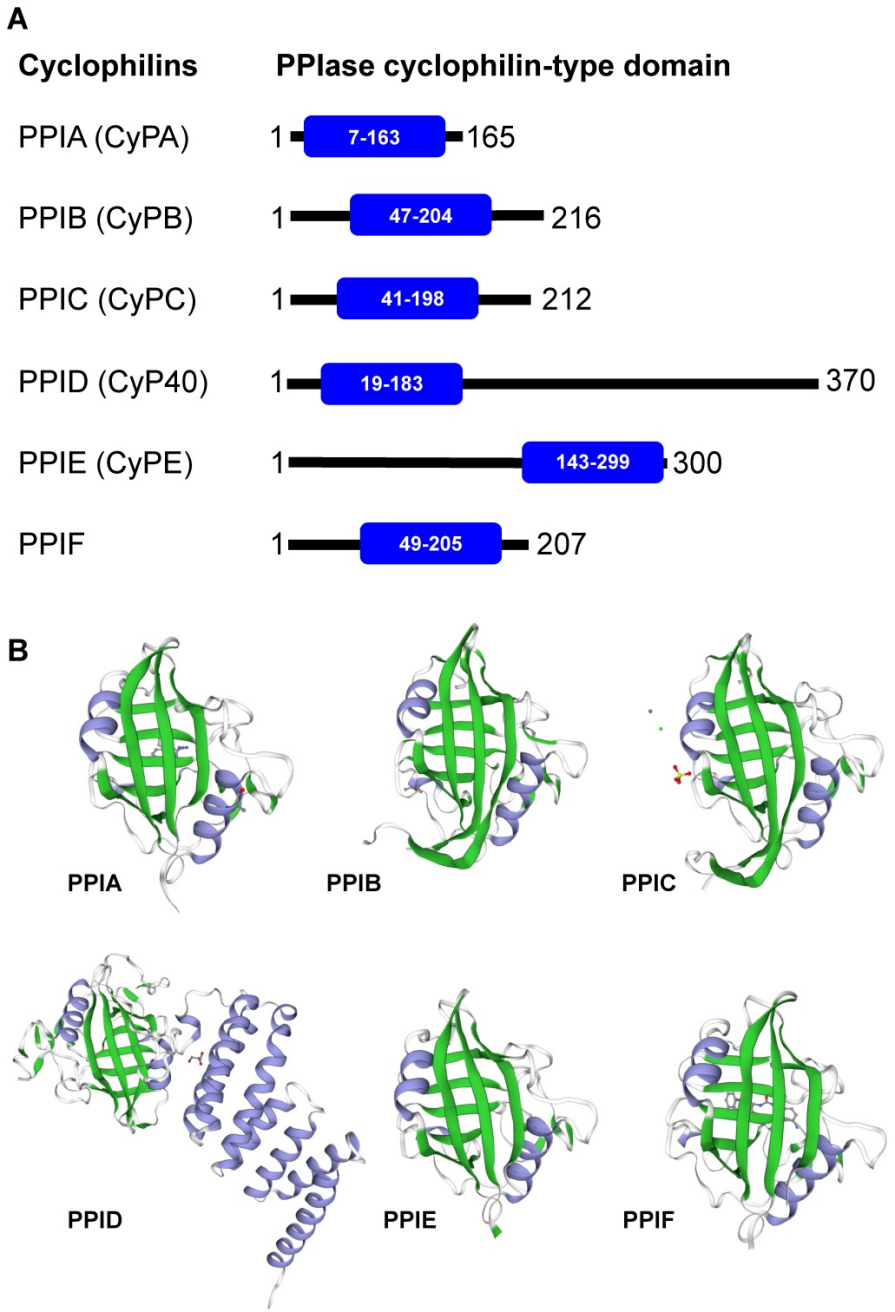
** Human cyclophilins.** Amino acid sequences, protein name, and/or description of human cyclophilins were obtained from The UniProt Knowledgebase (UniProtKB) database (*https://www.uniprot.org/*). **(A):** Alignment of human cyclophilins together with the PPIase cyclophilin-type domain in each cyclophilin. **(B):** The 3D structure of individual cyclophilins generated using SWISS-MODEL template library (*https://swissmodel.expasy.org/templates/*), including PPIA:PDB (4n1s), PPIB:PDB (1cyn), PPIC:PDB (2esl), PPID:PDB (1ihg), PPIE:PDB (2r99), and PPIF:PDB (5ccs). Their β-sheet and α-helix are labelled with green and greyish purple, respectively. Note that the unique structure of the cyclophilin protein family consists of 8 β-strands and 2 α-helices.

Cyclophilins are members of the immunophilin protein family, which is characterized by a signature domain containing PPIase activity 10. They are ubiquitously expressed in prokaryotes and eukaryotes, ranging from bacteria, fungi, insects, plants and mammals 11. Additionally, cyclophilins are widely expressed across many different organs 10, 12**.** The major forms of cyclophilins reported in humans include CyPA (PPIA), CyPB (PPIB), CyPC (PPIC), CyP40 (PPID), CyPE (PPIE), and PPIF 3. Herein, we avoid using an acronym CyPD, which is somewhat confusing as it can refer to PPID (encoded by the *PPID* gene on chromosome 4) and/or PPIF (encoded by the *PPIF* gene on chromosome 10). Mapping of the PPIase cyclophilin-type domain in these cyclophilins and their 3D structures are illustrated in **Figures 1A and 1B**, respectively. Most of the cyclophilins (CyPA, CyPB and CyPC) are found in cytoplasm and extracellularly, while CyPE is found in nuclear compartment and PPIF is identified as a mitochondrial cyclophilin 3. Among them, CyPA is the most abundant cyclophilin accounting for about 0.1-0.6**%** of total protein in the cytoplasmic compartment 12.

**Figure 2:**
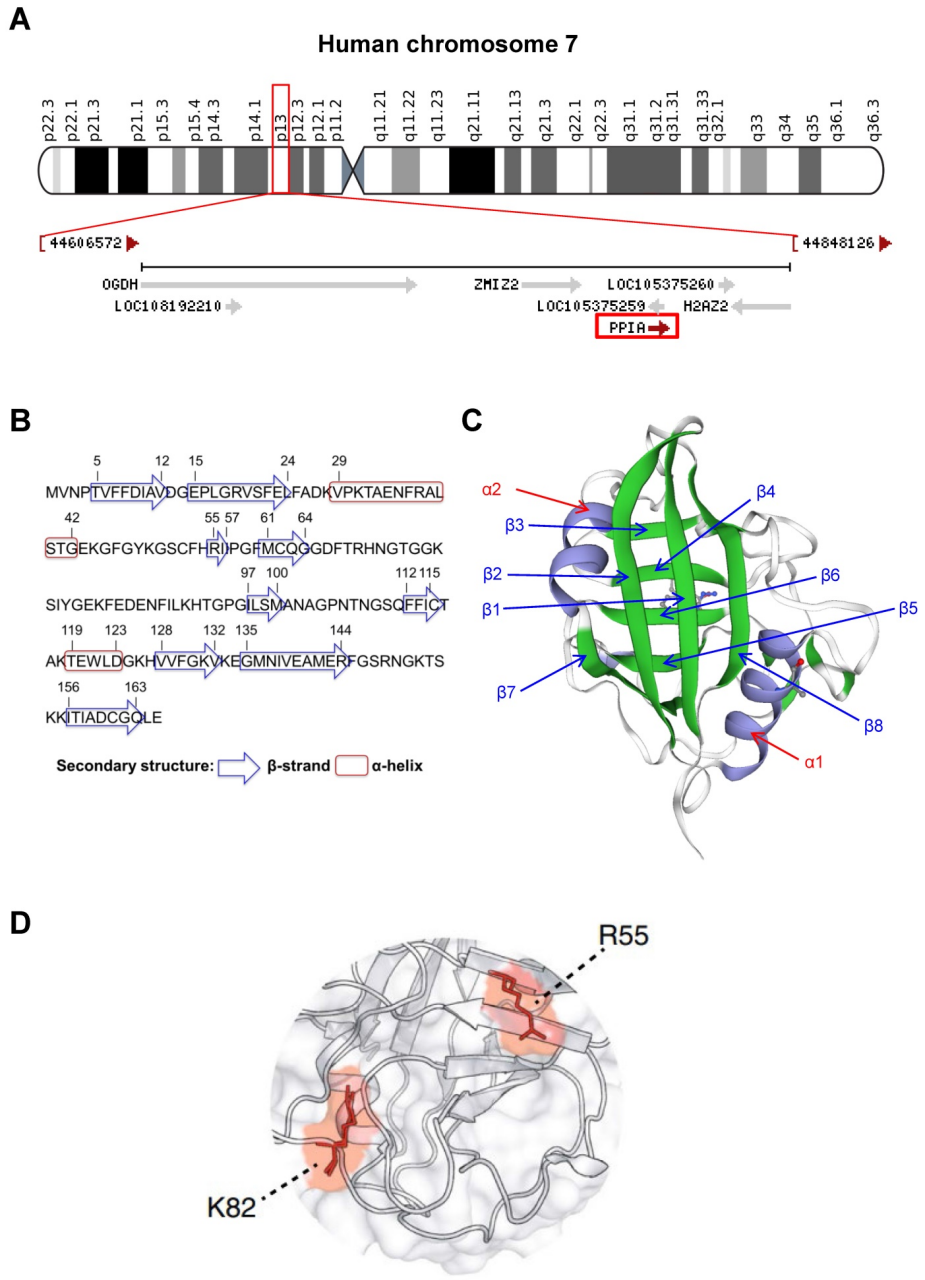
** General structural biology of CyPA. (A):** Mapping of location of *PPIA* gene on chromosome 7 at location 7p13 (NC_000007.14: 44,795,960-44,803,117) as indicated in the red box.** (B):** Amino acid sequence of CyPA. The residues that form the secondary structure (β-strand and α-helix) are labeled. **(C):** The 3D structure of CyPA with 8 β-strands and 2 α-helices. **(D):** The key amino acids, R55 and K82, that play crucial role in CyPA-mediated *cis/trans* isomerization (adopted from Ref. 13 that is licensed under a Creative Commons Attribution 4.0 International License, which permits use, sharing, adaptation, distribution, and reproduction in any medium or format).

CyPA is encoded by *PPIA* gene on chromosome 7 at location 7p13 (NC_000007**.**14**:** 44,795,960-44,803,117) **(Figure 2A).** This protein belongs to the cyclophilin**-**peptidyl prolyl isomerase**-**like family and comprises 165 amino acids with 8 β**-**strands and 2 α**-**helices **(Figures 2B and 2C)**. The 3D structure of the CyPA PPIase isomerization active site has been elucidated elsewhere, and the key residues have been characterized **(Figure 2D)**. The study has shown that arginine at the 55^th^ residue (R55) and lysine at the 82^nd^ residue (K82) are important for catalytic activity of CyPA-mediated *cis***/***trans***-**isomerization 13. This has been confirmed by alanine (A) substitutions at these two positions that lead to demolition of the catalytic function of CyPA 13.

Besides the PPIase isomerization function, CyPA can be secreted to the extracellular compartment to mediate chemotactic effects 14-16. This action occurs via specific binding of CyPA to a type I integral membrane glycoprotein, CD147 14-16. Proline and glycine residues at positions 180 and 181 (P180 and G181) of the CD147 extracellular domain are the key amino acids that mediate CyPA-CD147 interaction 17**.** Nonetheless, another later study has reported that P211 (instead of P180) of CD147 transmembrane domain is a critical residue for such binding 18. This has been supported by evidence demonstrating that P211A mutation drastically reduces CD147-derived peptide and CyPA interaction 19**.** Even though the results obtained from these studies are contradictory, CD147 evidently binds CyPA, while the precision of key residues responsible for such CyPA-CD147 interaction still needs further elucidations. On the CyPA side, its three amino acids, including R69, H70 and T107, have been identified as the key residues that play important role in CD147 binding 18**.**

## 3. Physiologic and pathophysiologic roles of CyPA in general

The physiologic function of CyPA**-**PPIase isomerase activity has been documented as a molecular chaperone that regulates protein folding, trafficking and activities 11, 20, 21**.** Several studies have also reported that CyPA is a multifunctional molecule with known major roles in cellular signaling, gene regulation, inflammation and apoptosis 21-23**.** The data obtained from VSMCs study has shown that reactive oxygen species (ROS) activate vesicle-associated membrane protein, resulting in secretion of CyPA via Rho-associate protein kinase (ROCK) pathway 24. ROCK is one of the serine/threonine kinases that serves as a key downstream effector of the small GTP-binding protein, RhoA, which regulates actin cytoskeleton organization and activates myosin II phosphorylation essential for transportation of CyPA toward plasma membranes 24.

CyPA undergoes various post-translational modifications 25, 26. CXCR4 (C-X-C motif chemokine receptor 4) signaling induces phosphorylation, while oxidative stress and angiotensin II promote acetylation of intracellular CyPA (iCyPA) 27, 28. The acetylated form of iCyPA is then secreted from the cells to extracellular space 28. Interestingly, acetylated extracellular CyPA (eCyPA), in turn, can display an autocrine effect to activate cellular functions 28. Moreover, acetylation at different lysine (K) residues in eCyPA can determine differential activities of eCyPA 28, 29. Generally, eCyPA has similar roles as of iCyPA (e.g., to induce inflammatory response and cell proliferation) 30, 31. Nevertheless, eCyPA also has some unique functions in activating apoptosis, cell migration, extracellular matrix (ECM) degradation, and ROS production 25, 30, 32, 33.

For pathophysiologic function, it has been documented that HK-2 renal cells under hyperglycemic condition have increased secretion of eCyPA, which in turn stimulates p38 mitogen-activated protein kinase (MAPK) pathway 34. Another study has also reported that eCyPA binds CD147, resulting in activation of ERK1/2 and p38 MAPK signaling pathways and cell proliferation 35. In concordance, an adhesion molecule secreted from *Mycoplasma genitalium* can induce secretion of eCyPA and its interaction with CD147 on urothelial cells, resulting in activation of extracellular signal-regulated kinase (ERK)/nuclear factor (NF)-κB pathway 36. As a result, cellular inflammatory response occurs together with production and release of proinflammatory cytokines and mediators, e.g., interleukin-1β (IL-1β), IL-6, tumor necrosis factor-α (TNF-α), and matrix metalloproteinase-9 (MMP-9) 36. Altogether, the accumulated evidence strengthens the role of eCyPA to evoke the inflammatory response.

CyPA has been reported to serve as a key factor in viral infections, including human immunodeficiency virus-1 (HIV-1) 37, hepatitis B virus (HBV) 38, hepatitis C virus (HCV) 39, and severe acute respiratory syndrome-coronavirus-2 (SARS-CoV-2) 40. The precise pathogenic roles for CyPA depend on type of the viral infection**.** For example, CyPA serves as an important intermediate proinflammatory cytokine that advocates the pathogenesis of SARS-CoV-2 infection through the CD147-dependent MAPK pathway 41**.** Also, plasma eCyPA level is greater in patients with severe coronavirus disease 2019 (COVID-19) as compared with those with milder forms of COVID-19 and healthy individuals 40.

Furthermore, many other diseases have been reported to be associated with the pleiotropic functions of CyPA**.** For example, elevation of eCyPA level correlates with cardiovascular diseases 42, rheumatoid arthritis 43, 44, and liver diseases 45. For kidney diseases, much greater details are provided in the following sections.

## 4. Roles of CyPA in kidney diseases

**Figure 3:**
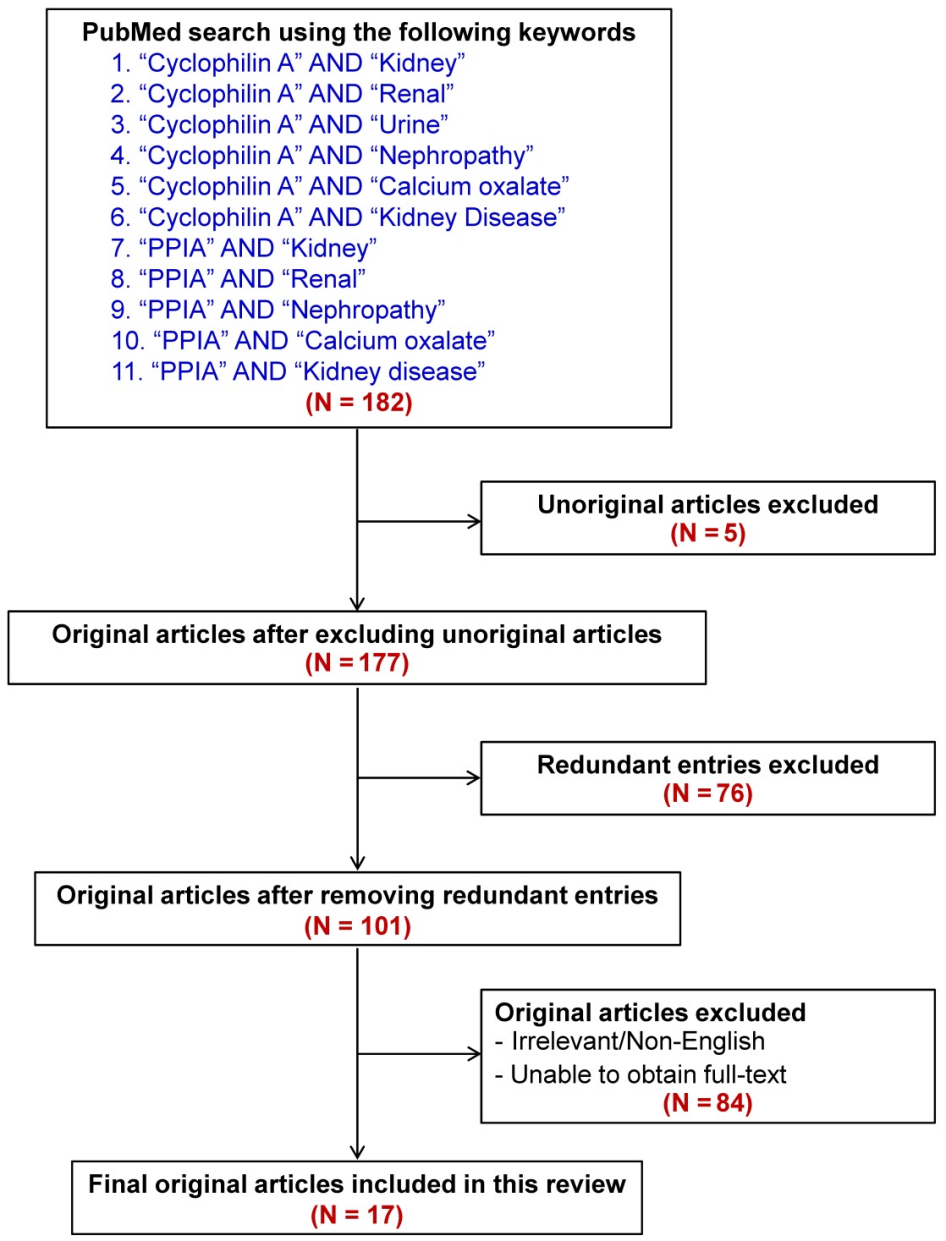
** Flow chart representing inclusion and exclusion literature search criteria to retrieve the articles for this review.** From a total of 182 articles initially retrieved using the well-defined keywords, 17 relevant articles were included for discussion in this review. Main findings reported in these articles are summarized in **Table 1**.

By using a pairwise searching method with the keywords indicated in **Figure 3**, a total of 182 articles were initially retrieved from PubMed literature search**.** The unoriginal, redundant, irrelevant, non-English, and fulltext-inaccessible articles were then excluded**.** Finally, there were a total of 17 articles qualified for discussion in this review (also summarized in **Table 1**). In addition, other articles that were not within the search criteria but their contents were related to our topic were also included for thorough discussion.

### 4.1. CyPA and diabetic nephropathy (DN)

Various cell types can secrete eCyPA under different conditions 21, 22. In diabetic patients, hyperglycemia and oxidative stress can stimulate the secretion of eCyPA from peripheral blood monocytes, resulting in a rise of plasma eCyPA level 46**.** Furthermore, a positive correlation has been found between plasma eCyPA level and age, blood sugar level and hemoglobin A1c (HbA1c) level 46**.** High glucose also activates signal transducer and activator of transcription 3 (STAT3)-CyPA interaction, which induces inflammation, oxidative stress, and apoptosis in podocytes 47. IL-37 is an anti**-**inflammatory and anti**-**immune response molecule**.** A recent study has shown that overexpression of STAT3 and CyPA leads to inhibition of the IL**-**37-mediated protective effects against podocyte injury induced by high glucose 47. These data on kidney cells are consistent with those obtained from human umbilical endothelial cells demonstrating that STAT3 promotes CyPA expression via binding to the STAT3-responsive element (SRE), a specific region in the CyPA gene (*PPIA*) promoter 48. Mechanistically, STAT3 forms a complex with co-factors and other transcription factors to regulate CyPA expression 48.

For almost two decades, we have known that CD147 is a membrane-bound glycoprotein that serves as a principal signaling receptor for circulating eCyPA 15, 19**.** It has been documented that eCyPA-CD147 binding can activate ERK/NF-κB pathway, leading to proinflammatory cytokines/chemokines release, leukocyte recruitment, and matrix overproduction 14, 49**.** Additional evidence has demonstrated that the complex of CyPA-CD147 is associated with inflammatory lesions 50. In the normal kidney, expression of CD147 is predominant in proximal and distal tubular cells**.** The increased expression of CD147 is found at the locales with infiltrating inflammatory cells as observed in kidney injury and lupus nephritis 51. Interestingly, plasma eCyPA and CD147 levels correlate with progression of DN in Type 2 diabetic patients 15.

In DN, which is a common disease worldwide 52, albuminuria is a traditional marker used for its clinical detection 53**.** However, albumin is absent in some DN patients who had already developed end**-**stage renal disease (ESRD) 54. Therefore, a higher sensitive biomarker for DN is urgently required**.** Tsai et al**.** 55 have reported, for the first time, the potential role of urinary CyPA (uCyPA) as a novel biomarker for early detection of DN**.** Their investigations in kidney cell lines, mesangial (MES-13) and tubular (HK-2) cells, have also shown that eCyPA can be secreted from both cell types**.** Furthermore, measurement of uCyPA level in DN outpatients has revealed 90**%** sensitivity and 72.7**%** specificity for its use to diagnose Stage 2 DN with a moderate to high ROC (receiver operating characteristic) curve for diagnostic power (AUC = 0.85) 55**.** However, coefficient of determination of uCyPA with urinary albumin/creatinine (ACR) ratio is very low (R^2^ = 0.054) 55. A study in an animal model has also shown that uCyPA level in 24**-**h urine is significantly higher in diabetic rats compared with the non**-**diabetic controls with 77.8% sensitivity, 67% specificity, 70% positive predictive value (PPV), 75% negative predictive value (NPV), and 0.778 AUC of ROC curve 56**.** Coefficient of correlation of uCyPA with ACR and 24-h urinary protein are both statistically significant (R = 0.426, *p* = 0.011 and R = 0.456, *p* = 0.043, respectively) 56**.** This result is consistent with the findings in another study by Tsai et al. 34, who have reported that uCyPA concentration increases 12**.**7-fold in the diabetic mice at eighth week. Moreover, uCyPA (68.3% sensitivity, 53.3% specificity, 74.5% PPV, 45.7% NPV, 0.856 AUC of ROC curve) and uCyPA/creatinine ratio (62.5% sensitivity, 93.3% specificity, 90.8% PPV, 70.4% NPV, and 0.830 AUC of ROC curve) both increase in Type 1 DM pediatric patients with microalbuminuria 57**.** These studies have concluded that uCyPA may serve as an effective biomarker for early DN detection due to its high sensitivity, high specificity and non-invasiveness 55-57.

### 4.2. CyPA and acute kidney injury (AKI)

AKI is a renal disorder recognized by the rapid loss of kidney function or kidney damage that rapidly develops within hours or days**.** The most common causes of AKI include acute tubular necrosis and prerenal azotemia 58. Kidney cells, particularly renal tubular epithelial cells, are prone to the damage and susceptible to intrinsic oxidative stress such as excessive inflammatory response and ischemia 59. Under such conditions, eCyPA serves as a key oxidative stress**-**induced secretory factor 60. eCyPA also serves as a chemotactic factor to induce leukocyte infiltration at the inflammatory locales 21, 61. The increase of eCyPA secreted from human proximal tubular epithelial cells (PTECs) is found after the cells are exposed to harmful agents 62. PTECs also secrete greater amount of eCyPA during cell death and/or kidney injury 63.

By the rise of eCyPA level, accumulation of leukocytes to the injured site is promoted and tissue damage is amplified. CyPA has been demonstrated to increase in the kidney of ischemia/reperfusion-induced AKI animal model, whereas knockdown of its gene (*PPIA*) reduces tissue inflammation 64. To address the pathogenic role of eCyPA in AKI, the effect of a cyclophilin inhibitor (GS**-**642362, which was derived from sanglifehrin A macrocycle 65) has been examined. The data have shown that GS**-**642362 has a protective effect against acute renal failure in the renal ischemia**/**reperfusion-induced AKI model in a dose**-**dependent manner 62. Such protective effect correlates with the decrease of tubular cell death via the reduction of neutrophil infiltration 62**.** These findings highlight the pathogenic roles of eCyPA in AKI and its potential to be used as a novel biomarker for early detection of AKI**.**

eCyPA is secreted from the damaged cells to the extracellular environment 63**.** Moreover, circulating eCyPA can filter freely through the glomerulus in the nephron**.** Therefore, a high level of uCyPA may reflect elevation of eCyPA secretion from the toxic renal cells and/or its increased glomerular filtration or leakage 63, 66. The increase of uCyPA in ischemia/reperfusion-induced AKI can thus serve as the biomarker independent from other functional and clinical parameters 63, 66. Also, it is evident that uCyPA may be used in complement with serum creatinine and other classical markers for AKI, e.g., neutrophil gelatinase**-**associated lipocalin (NGAL). Moreover, it can be used for the diagnosis of AKI in patients who do not match the AKI functional impact**-**based diagnostic criteria 59, 66.

In addition to CyPA, other cyclophilins/immunophilins are also involved in AKI and deserve discussion here. PPIF is a mitochondrial protein that acts as an essential regulator of the mitochondrial permeability transition pore (mPTP) 67, 68. Apoptotic stimuli can induce PPIF to form a complex with p53, leading to mPTP opening, mitochondrial swelling, leakage of cytochrome c into cytoplasm, and tubular cell death 69, 70. A recent study has proven for the first time that PPIF contributes to acute tubular necrosis and AKI induced by high dose of plant-derived nephrotoxic agent, aristolochic acid 70. By contrast, the loss of renal functions, tubular cell damage and death, and neutrophil infiltration are improved in the *PPIF^-/-^* mice 70. These findings are consistent with those reported in an earlier study showing that proximal tubule-specific *PPIF*-knockout mice are protective from cisplatin-induced fatty acid β-oxidation (FAO) and AKI 71. Such protective mechanism is mediated by PPIF-peroxisome proliferator-activated receptor-α (PPARα) complex formation within mitochondria. This complex suppresses nuclear transcription of PPARα-regulated FAO genes during cisplatin-induced AKI 71. Taken together, although CyPA and PPIF promote AKI by different mechanisms, both of them serve as the promising therapeutic targets for management of AKI.

FK506-binding protein 12 (FKBP12) is another immunophilin being a primary target for two structurally related drugs, FK506 and rapamycin 72. Binding of FKBP12 with FK506 inhibits bone morphogenetic proteins (BMPs)-related signaling pathway 73. Among several BMPs, BMP7 is necessary for development and homeostasis of the kidney 74. In contrast to CyPA and PPIF, BMP7 preserves kidney function in an animal model of AKI by restoring PTECs function and inflammatory response 75, 76. The binding of FKBP12 to FK506 can be inhibited by using a FK506 analog, oxtFK, thereby promoting the BMP7 activity to prevent AKI induced by ischemia**/**reperfusion 73. These data highlight the promising role of FKBP12 as a new therapeutic target for treatment of AKI.

### 4.3. CyPA in chronic kidney disease (CKD) and renal fibrosis

CKD is characterized by the presence of chronic morphological and functional disorders in the kidney that can progress to ESRD 77-79**.** General pathological processes of CKD and many other diseases are frequently associated with inflammation**.** Both local and systemic inflammatory responses are involved in these CKD processes**.** The presence of the cardiovascular mesh system allows the inflammatory mediators to travel throughout the body, from one organ to another**.** It has been shown that CKD is associated with peripheral arterial occlusive disease (PAOD) with a high incidence 80. PAOD is an atherosclerotic disease with lesions in the lower extremities and intermittent claudication as the major and classical clinical manifestations**.** A previous study has shown that CyPA plays role in the pathogenesis and progression of PAOD 81. CyPA is secreted by VSMCs in response to oxidative stress and promotes the development of atherosclerosis in many ways 81**.** CyPA can also facilitate migration and proliferation of VSMCs, stimulate proinflammatory cytokine pathways in endothelial cells, exert chemotactic effects, and enhance ROS production 81-84**.** The inflammatory mediators generated in the vascular system can definitely enter into the kidney as well as other organs and affect the cells that are exposed to them**.** It has been documented that a high serum level of eCyPA may be related to the decline of renal function 81. Furthermore, markedly elevated plasma eCyPA positively correlates with systemic inflammation markers, such as high-sensitivity C-reactive protein, IL-6 and TNF-α, in ESRD patients undergoing hemodialysis and peritoneal dialysis 85.

It is interesting that PTECs also serve as the non**-**professional antigen**-**presenting cells **(**APCs**)** in the kidney tissue that play role in modulation of immune responses 86**.** This gives rise to the question that whether the immune modulation in kidney injury occurs via the effect of the non**-**professional APCs function of PTECs or the effect of CyPA. Alternatively, both induction pathways may occur and cooperate**.** The general pathological processes of CKD normally involve both local and systemic inflammatory responses 79. Under the CKD environment, eCyPA affects the impairment of renal function through the vascular mesh networks**.** The limitation of tissue regeneration after cellular injury in the kidney can lead to the progressive decline of renal function and ultimately ESRD 79.

Renal fibrosis is a key determinant and prognostic marker for the chronic progression of kidney failure**.** The molecular mechanisms of CyPA in promoting tubulointerstitial fibrosis after kidney injury have been investigated 62**.** Following tissue injury, eCyPA promotes leukocyte recruitment and inflammatory cascade 62**.** These data underline the important role of CyPA in the pathogenesis of renal fibrosis**.** Moreover, the eCyPA level is associated well with the degree of renal fibrosis 87. Therefore, eCyPA also serves as a potential biomarker for CKD and renal fibrosis.

There is *in vitro* evidence suggesting that inhibition of CyPA activity significantly decreases ECM protein production and accumulation that may exert a therapeutic effect on renal fibrosis 87**.** Although convincing, validation in clinical setting is required. Since fibrosis is not limited to just one organ, a common fibrotic pathway has been thought to exist 88. Interestingly, caveolin-1 (a vesicular transport regulator) has been shown to reduce CyPA-induced ROS overproduction in an animal model of hypercholesterolemia-associated atherosclerosis and renal damage 84. Similar effects of CyPA inhibition have been found also in cardiac diseases. For example, inhibiting the eCyPA activity significantly reduces inflammation and myocardial fibrosis 89. In addition, a recent study has also reported the pathogenic role of PPIF in the development of renal fibrosis, as *PPIF* gene deletion minimizes tubular cell apoptosis, protects peritubular capillary loss, and reduces kidney inflammation in the obstructive kidney 90. Interestingly, GS-642362 (a potent CyPA inhibitor as mentioned above) also inhibits PPIF 62. In addition to ischemia**/**reperfusion-induced AKI, GS-642362 also reduces cell death, macrophage infiltration, and fibrotic development in the unilateral ureteric obstruction (UUO) model of renal fibrosis 62. However, the degree of such protection in the UUO model is less than that in the ischemia/reperfusion-induced AKI model 62. Therefore, both eCyPA and PPIF are involved in the pathogenesis of renal fibrosis and serve as the new therapeutic targets for treatment of CKD and renal fibrosis.

### 4.4. CyPA in nephrotoxicity associated with organ transplantation

In organ transplantation, CsA usually serves as a major immunosuppressive drug**.** It is widely used in tissue and organ transplantation to prevent rejection and acute graft-versus-host disease (aGVHD) 7. This first-line therapy involves the ability of the CsA-CyPA complex to bind calcineurin, resulting in immune suppression by reduced production of IL**-**2 and other cytokines, as well as inhibition of T-cell activation 7, 91. Nonetheless, clinical use of CsA has many adverse events, including nephrotoxicity, neurotoxicity, malignancy risk and infection 7, 92, 93**.** It has been reported that long**-**term exposure to CsA induces renal tubular cell atrophy and interstitial fibrosis 94, 95**.** CsA promotes interstitial ECM accumulation by a combination of suppressed activity of matrix degradation enzyme, enhanced synthesis of collagen from renal cortical fibroblasts, induction of autocrine insulin**-**like growth factor**-**I (IGF-I) secretion and function, and increased secretion of transforming growth factor-β1 (TGF-β1) from PTECs. Modulation of the cytokine networks has been shown to play an important role in the tubulointerstitial pathology 94, 95**.**

Indeed, CyPA also serves as a key modulator of CsA action. A recent study has demonstrated that knockdown of gene encoding CyPA (*PPIA*) in renal cells increases the unfolded protein response (UPR) similar to the effects of CsA treatment, which suppresses the CyPA chaperone function, leading to ER stress, UPR and renal epithelial cell apoptosis 96. Modifying CyPA and/or UPR may help to reduce nephrotoxicity associated with CsA in renal transplantation 96. Another study has reported that polymorphism (-11G/C) on *PPIA* promoter is related to the nephrotoxicity after renal transplantation by affecting the *PPIA* gene expression 97**.** Therefore, monitoring this *PPIA* gene polymorphism in organ transplant patients may help to prevent secondary nephrotoxicity before undergoing serious progression**.**

## 5. Proposed pathogenic mechanisms of CyPA in kidney diseases

**Figure 4:**
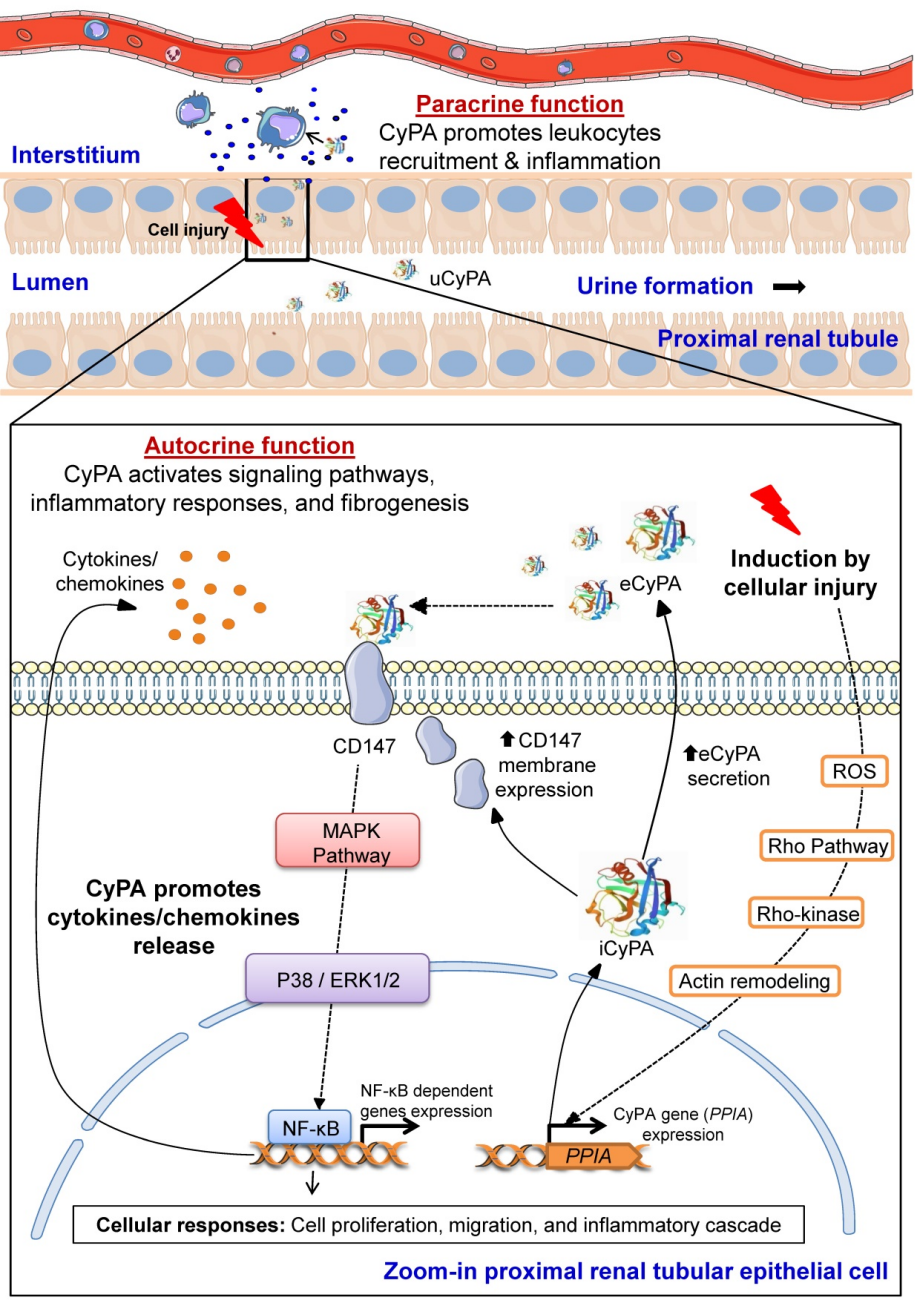
**Proposed pathogenic mechanisms of CyPA in kidney diseases.** During cellular injury, CyPA expression and secretion are induced by various stimuli, e.g., oxidative stress, hypoxia, infection, inflammation, hyperglycemia and mechanical stretch, via the Rho-dependent pathway. The downstream cascade is associated with Rho kinase and actin remodeling mediators. Intracellular CyPA (iCyPA) can be secreted (eCyPA) from the cells into the extracellular compartment and performs paracrine and autocrine functions. For the autocrine function, the binding of eCyPA to CD147 activates MAPK pathway via p38, ERK1/2 and NF-κB, leading to cell proliferation, migration and inflammatory cascade. For the paracrine function, eCyPA secreted from the injured cells promotes accumulation and activation of leukocytes, such as neutrophils, monocytes and T-cells, which subsequently mediate tubular cell necrosis, interstitial inflammation, fibrogenesis and impaired kidney function. Finally, the increase in circulating eCyPA and uCyPA serves as a promising biomarker for many kidney diseases.

ROCK pathway has been demonstrated to play roles in VSMCs functions and is hence associated with cardiovascular diseases 98, 99**.** Previous studies have shown that up-regulation of CyPA at aortic aneurysm and atherosclerotic plaques is mediated by ROCK activity 99, 100**.** This data is consistent with that reported from another study demonstrating that CyPA is a ROS**-**related protein that is secreted by VSMCs under the RhoA**/**Rho**-**kinase activation 99, 101**.** This raises the possibility that other cells that express CyPA may also behave the same under similar conditions**.** On this basis, we have proposed herein the pathogenic mechanisms of CyPA in kidney diseases, especially at PTECs, in which CyPA expression is highly predominant**.** The schematic representation of CyPA-induced pathogenic mechanisms of kidney diseases is shown in **Figure 4**.

In response to stimuli such as oxidative stress, inflammation, hypoxia, infection, hyperglycemia and mechanical stretch, expression and transcription of *PPIA* gene encoding CyPA is induced via Rho**-**kinase activation. iCyPA subsequently plays a role as a chaperone and regulator for protein trafficking and activity. Additionally, iCyPA induces membrane expression of CD147 55. CyPA can be also secreted (eCyPA) via the vesicular secretion pathway into the extracellular space and performs both paracrine and autocrine functions 55. The binding of eCyPA to CD147 mediates cellular signaling cascade via MAPK pathway involving p38, ERK1**/**2 and NF**-**κB, which further trigger cell proliferation, migration and inflammatory response**.** Subsequently, proinflammatory cytokines/chemokines are released and stimulate the downstream cellular signals that affect the progression of tubular injury, interstitial inflammation and fibrogenesis 55. Finally, overproduction of eCyPA results in the increase of circulating eCyPA and uCyPA that can be used as promising biomarkers for early diagnosis and prognosis of several kidney diseases 55.

## 6. Conclusions

CyPA is a multifunctional molecule that plays important roles as a key factor in many pathological conditions. Herein, we highlight its theranostic roles in various kidney diseases, including DN, AKI, CKD, renal fibrosis, and nephrotoxicity associated with organ transplantation**.** eCyPA is secreted from the cells via the vesicular secretion pathway to exert paracrine and autocrine effects. eCyPA can bind to CD147 and subsequently trigger MAPK signaling pathway via downstream p38, ERK1/2 and NF-κB, resulting in cell proliferation, migration, inflammatory cascades, progression of tubular injury, interstitial inflammation and fibrogenesis. Circulating eCyPA can filter freely through the glomeruli**.** A high level of uCyPA may reflect an elevation of plasma eCyPA level and/or its increased secretion by the damaged renal tubular cells**.** Therefore, uCyPA and plasma eCyPA serve as the promising biomarkers for diagnostics and prognostics in various kidney diseases**.** Since CyPA function has a high impact on the pathogenesis of several kidney diseases, CyPA may serve as a new therapeutic target, and utilization of a clinically safe CyPA inhibitor can be considered as an alternative therapeutic approach for future management of these kidney diseases.

## 7. Future perspectives

It is evidently clear that CyPA plays significant theranostic roles in various kidney diseases. Nevertheless, its roles in kidney diseases seem to be under-investigated. In addition to DN, AKI, CKD, renal fibrosis, and nephrotoxicity associated with organ transplantation, the pathogenic and theranostic roles of CyPA should be more extensively elucidated in several other kidney diseases.

Note that most of the previous studies have reported the potential roles of uCyPA and plasma eCyPA as the new diagnostic/prognostic markers for kidney diseases. However, only few of them have compared their sensitivity, specificity, PPV, NPV, accuracy, etc. with other conventional tests. Therefore, future research of CyPA should also focus on such comparative analyses with the gold standards. Additionally, the use of different forms of CyPA (uCyPA and plasma eCyPA) as the diagnostic/prognostic markers should be validated in large patient cohorts of these kidney diseases aiming toward their clinical applications at the bedside.

Special attention for future CyPA research should be paid to its promising role as a new therapeutic target for treatment and/or prevention of kidney diseases. CyPA inhibitors exert therapeutic effects in several disease entities. In addition to GS-642362 with a potential therapeutic role in AKI and renal fibrosis as mentioned above, several other CyPA inhibitors deserve further investigations in kidney diseases. Because plasma eCyPA level correlates with kidney disease progression, eCyPA seems to be a more potent pathogenic mediator as compared with iCyPA 102. MM218, a CsA derivative in which its chemical group was modified to be cell-impermeable, selectively inhibits eCyPA but does not affect iCyPA 103, 104. MM218 has been shown to effectively decline inflammation by blocking eCyPA-mediated leukocyte accumulation in allergic lung inflammation in a murine model 103. In amyotrophic lateral sclerosis (ALS), which is frequently unresponsive to treatment, MM218 has been shown to protect motor neurons and concomitantly reduces MMP-9 production 104. MM218 also reduces NF-κB activation in the spinal cord of the ALS mice 104.

MM284 is another eCyPA-specific cell-impermeable inhibitor. A previous study has demonstrated that MM284 blocks eCyPA-induced migration and adhesion of monocytes and reduces MMP-9 expression in cardiac tissue of animals with myocarditis 102. Additionally, CRV431 (another CsA derivative) potently inhibits not only CyPA but also other cyclophilins and has been demonstrated to decrease liver fibrosis and tumor masses in rodent models 105. Furthermore, beneficial effects of many other non-immunosuppressive CsA derivatives, such as NIM811, SCY-635 and STG-175, have been reported in other diseases, particularly viral infections 106-108. Although not yet tested, these and many other CyPA inhibitors may also exert therapeutic effects for kidney diseases and, therefore, deserve further extensive investigations.

It should be noted that the inhibitors used in several of previous studies mentioned above may not be specific only to CyPA (as they also inhibit other cyclophilins), and their chemical structures or characteristics are not well defined. Therefore, further developments of the specific CyPA inhibitors with precise information of their chemical structures, characteristics and kinetics are required. Among the cyclophilin inhibitors mentioned above, NIM811 is associated with modest increases in bilirubin and triglyceride as well as thrombocytopenia 109. On the other hand, MM218 and CR431 seem to have safer profiles for clinical use 103, 105. Although with potential therapeutic roles in kidney diseases, the adverse events of these CyPA inhibitors require further monitoring.

As mentioned above, a recent study has demonstrated that *PPIA* gene knockout can prevent ischemia/reperfusion-induced AKI, but is not protective against UUO-induced renal fibrosis 64. A more recent study using small interring RNA (siRNA) specific to *PPIA* gene has demonstrated the induction of UPR by *PPIA*-knockdown in renal cells similar to the effects of CsA treatment to suppress CyPA function 96. Therefore, knockout/knockdown of the *PPIA* gene encoding CyPA is another interesting aspect for future research on the theranostic roles of CyPA in kidney diseases to explore. Not only the specific effects on CyPA and disease outcome, their off targets should be also characterized.

Taken together, all of these data strongly indicate that CyPA serves as a promising therapeutic target for new drug development and should be more seriously concerned for its applications in treatment and prevention of kidney diseases.

## Figures and Tables

**Table 1:** Summary of main findings in previous studies on roles of CyPA in kidney diseases.

Kidney disease	Reference*	Publication date	Main findings	Type of sample(s)	CyPA role in diagnostics/ prognostics	CyPA role in therapeutics
**Diabetic nephropathy (DN)**	Ramachandran S, et al. 46	2014	Level of circulating extracellular CyPA (eCyPA) significantly increases in diabetic patients.	Human plasma.	Yes	-
	Tsai SF, et al. 55	2015	The first report demonstrating that urinary CyPA (uCyPA) serves as a new biomarker for early detection of DN**.**	Human urine. MES-13 cells. HK-2 cells.	Yes	-
	Tsai SF, et al. 34	2016	uCyPA is a sensitive marker for early detection of DN**.**	Mouse urine. MES-13 cells. HK-2 cells.	Yes	-
	Chiu PF, et al. 15	2018	Plasma eCyPA and CD147 levels correlate with progression of DN in Type 2 diabetic patients.	Human plasma.	Yes	-
	Zhang X, et al. 47	2020	Overexpression of STAT3 and CyPA leads to podocyte injury induced by high glucose.	Mouse podocytes.	Yes	-
	El**-**Ebidi AM, et al. 56	2020	uCyPA level in 24**-**h urine is significantly higher in diabetic rats compared with non**-**diabetic controls**.**	Rat urine.	Yes	-
	Salem NA, et al. 57	2020	uCyPA and uCyPA**/**Cr ratio increase in Type 1 DM pediatric patients with microalbuminuria**.**	Human urine.	Yes	-
**Acute kidney injury (AKI)**	Lee CC, et al. 66	2019	eCyPA can be used for postoperative AKI detection in cardiac surgery patients**.** Circulating eCyPA and uCyPA levels markedly increase in AKI compared with non**-**AKI group**.**	Human serum. Human urine.	Yes	-
	Leong KG, et al. 64	2020	CyPA increases in the kidney during renal ischemia/reperfusion-induced AKI. Knockdown of its gene (*PPIA*) reduces tissue inflammation.	Mouse kidney. Mouse primary tubular cells.	Yes	-
	Leong KG, et al. 62 #	2020	CyPA inhibitor (GS-642362) has a protective effect against acute renal failure in the renal ischemia/reperfusion-induced AKI model in a dose-dependent manner. It decreases tubular cell death via reduction of neutrophil infiltration.	Mouse kidney. Mouse plasma. Mouse primary tubular cells.	-	Yes
	Cabello R, et al. 63	2021	Renal tubular cells secrete greater amount of eCyPA during different cell death pathways**.** Increasing uCyPA serves as the biomarker of ischemia/reperfusion-induced AKI independent from other parameters of kidney function**.**	HK-2 cells. MCT cells. Human urine.	Yes	-
**Chronic kidney disease (CKD) and renal fibrosis**	Liu MC, et al. 81	2015	The level of serum eCyPA may be associated with impaired renal function in CKD**.** eCyPA released from kidney cells or other cell types under the CKD environment promotes atherosclerosis**.**	Human serum.	Yes	-
	Chen YH, et al. 84	2016	CyPA is a mediator for ROS production**.** Caveolin-1 inhibits CyPA-induced ROS overproduction.	Rabbit kidney. Rabbit serum.	-	Yes
	Jin K., et al. 85	2017	Markedly elevated plasma eCyPA positively correlates with systemic inflammation markers, such as high-sensitivity C-reactive protein, IL-6 and TNF-α in ESRD patients.	Human plasma.	Yes	-
	Dihazi GH., et al. 87	2020	Secretion of eCyPA is associated with renal fibrosis**.** Inhibition of CyPA activity decreases ECM protein production and accumulation.	Mouse kidney. TK173 cells. TK188 cells.	Yes	Yes
	Leong KG, et al. 62 #	2020	CyPA inhibitor (GS**-**642362) also inhibits PPIF. GS-642362 decreases tubular cell death, macrophage infiltration, and renal fibrosis in unilateral ureteric obstruction (UUO) animal model.	Mouse kidney. Mouse plasma. Mouse primary tubular cells.	-	Yes
**Nephrotoxicity associated with organ transplantation**	Moscoso-Solorzano GT, et al. 97	2008	Polymorphism (-11 G/C) on *PPIA* promoter is associated with nephrotoxicity after renal transplantation.	Human (EDTA-preserved) blood.	Yes	-
	Yilmaz DE, et al. 96	2022	*PPIA* knockdown increases the unfolded protein response (UPR) similar to the effects of CsA treatment. Modifying CyPA and/or UPR may help to reduce nephrotoxicity associated with CsA in renal transplantation.	HEK-293 cells. HRPTEpCs primary cells. Rat proximal tubules.	-	Yes

* See details of search parameters and criteria in **Figure 3**. # Both AKI and CKD/renal fibrosis were investigated in this study.
